# A randomized double blind comparison of atosiban in patients with recurrent implantation failure undergoing IVF treatment

**DOI:** 10.1186/s12958-022-00999-y

**Published:** 2022-08-19

**Authors:** Chuan Ling Tang, Qi Yue Li, Feng Lian Chen, Chen Ting Cai, Yue Yan Dong, Yuan Yuan Wu, Jian Zhi Yang, Mei Zhao, Feng Li Chi, Ling Hong, Ai Ai, Miao Xin Chen, Kun Ming Li, Xiao Ming Teng, Zhi Qin Chen

**Affiliations:** Shanghai First Maternity and Infant Hospital, School of Medicine, Tongji University, Shanghai, 200092 China

**Keywords:** Atosiban, Recurrent implantation failure, Fresh embryo transfer, Live birth rate, Endometrial peristalsis

## Abstract

**Background:**

Patients with recurrent implantation failure (RIF) may have more uterine contractions. Several observational studies suggested that atosiban administration around embryo transfer resulted in higher pregnancy rates in RIF patients. This study aimed to evaluate the effect of atosiban given before fresh embryo transfer on pregnancy outcomes of women with RIF.

**Methods:**

A prospective, randomized, double-blind controlled clinical trial was performed in IVF center of Shanghai First Maternity and Infant Hospital. According to a computer-generated randomization list, 194 infertile women with RIF received fresh embryo transfer between July 2017 and December 2019 were randomly allocated into the atosiban (*n* = 97) and the placebo (*n* = 97) groups. Women in the treatment group received atosiban intravenously about 30 min before embryo transfer with a bolus dose of 6.75 mg over one minute. Those in the placebo group received only normal saline infusion for the same duration.

**Results:**

There was no significant difference in the live birth rate between the atosiban and placebo groups (42.3% *vs* 35.1%, *P* = 0.302, RR = 1.206 (0.844–1.723)). No significant differences were found between the two groups in the positive pregnancy test, clinical pregnancy, ongoing pregnancy, miscarriage, multiple pregnancy, ectopic pregnancy and implantation rates. Similar results were found when stratified by the number of embryos previously transferred, number of previous failed embryo transfers, frequency of endometrial peristalsis on embryo transfer day (≥ 3 waves/min) or serum estradiol (E_2_) on the day of hCG above the median level. And, there was no correlation between the serum E_2_ level on the day of hCG and the frequency of endometrial peristalsis on embryo transfer day. The frequency of endometrial peristalsis on embryo transfer day, total FSH/HMG dosage and duration were the significant factors which independently predicted the likelihood of a live birth.

**Conclusions:**

These results suggested that atosiban treatment before fresh embryo transfer might not improve the live birth rate in RIF patients.

**Trial registration:**

The study had been approved by the Institutional Review Board of the hospital (2017 ethics No.43) and was registered under Clinicaltrials.gov with an identifier NCT02893722.

## Introduction

IVF-ET is an important treatment options for the infertile couples. However, recurrent implantation failure (RIF) becomes a difficult problem in the clinical practice of assisted reproductive technology (ART), and there is no universally accepted definition. It is generally believed that RIF refers to failure to achieve a clinical pregnancy after transfer of at least four good-quality embryos in a minimum of three fresh or frozen cycles in a woman under the age of 40 years [1]. The incidence of RIF is about 10–20%, and the causes of RIF in IVF are varied and complex, including poor embryo quality, abnormal uterine cavity, reduced endometrial receptivity, and impaired immune functions [2].

Embryo implantation is a complicated process. Embryo quality and intrauterine environment are the two main factors which affect embryo implantation [3]. The ideal uterine conditions that allow embryo implantation require adequate blood supply to endometrium and moderate uterine contractions [4]. Excessive uterine contractions can affect embryo implantation, even cause the embryo to be discharged from the uterine cavity [5]. An increased frequency of endometrial peristalsis in frozen-thawed cycle of RIF women has been recently confirmed [6, 7]. Therefore, inhibiting uterine contractions may be an effective measure to improve the success rate of assisted pregnancy in RIF women.

Atosiban is a combined oxytocin/vasopressin V1A antagonist, which is registered for clinical use in women suffering from imminent preterm labour. The mechanism of atosiban is to compete with oxytocin to its receptors located in the myometrium, decidua and fetal membranes, and reduce the efficacy of oxytocin and the level of calcium ions on muscle cells, thereby inhibit uterine contractions [8]. The first case of clinical use of atosiban in IVF was reported by Pierzynski et al. [9]. Atosiban was administered to a 42-year-old patient who had previously undergone eight transfers of 12 good quality embryos and she conceived in that cycle. In a prospective cohort study, Lan et al. [6] showed that atosiban may benefit women with RIF undergoing transfer of cryopreserved embryos in a hormonal replacement cycle. The only multi-center RCT involving 800 IVF patients by Ng et al. [10] showed that there was no significant difference in clinical pregnancy rate and live birth rate between the atosiban and the placebo groups for a general population of patients undergoing IVF. However, subgroup analysis in this study revealed no difference in all pregnancy outcomes between the two groups in a repeated cycle. Since patients undergoing repeated IVF cycles obviously cannot be compared with those with RIF, therefore, their results should not be extrapolated to RIF management.

For the above reason, there is clearly a need for a randomized double- blind study to compare the pregnancy outcomes between women receiving atosiban and placebo around embryo transfer in women with RIF. The hypothesis in the present study was that the live birth rate was significantly higher after the use of atosiban in women with RIF undergoing IVF treatment.

## Materials and methods

### Study population

This prospective, double-blind study was conducted in Shanghai First Maternity and Infant Hospital between July 2017 and December 2019. Consecutive women attending the center for IVF were screened and recruited if they fulfilled the selection criteria. The inclusion criteria included: (i) less than 40 years of age; (ii) failure to achieve a clinical pregnancy after transfer of at least four good-quality embryos in a minimum of three fresh or frozen cycles; (iii) use of gonadotropin-releasing hormone (GnRH) agonist or antagonist protocol for ovulation induction; (iv) endometrial thickness ≥ 8 mm on day of hCG; (v) normal uterine cavity shown on hysterosalpingogram or hysteroscopy. (vi) one or more D3 good-quality embryos on the day of embryo transfer. Women were excluded if they had: (i) use of donor eggs/sperm; (ii) hydrosalpinges shown on scanning and not treated; (iii) moderate or severe endometriosis; (iv) an abnormal chromosome in either or both partners; (v) a congenital uterine anomaly; (vi) blastocyst transfer; (vii) unclear information of previous transfer cycles.

All women were fully counselled and informed written consents were obtained prior to participation. All participants are voluntarily joined this study which means no monetary benefit was paid during the recruiting. The study had been approved by the Institutional Review Board of the hospital (2017 ethics No.43) and was registered under Clinicaltrials.gov with an identifier NCT02893722.

### Ovarian stimulation and IVF

Women started ovarian stimulation using either the long agonist or antagonist protocol. For the long protocol, 1.25 mg GnRH agonist (Triptorelin acetate, Diphereline, Ipsen Pharma Biotech, France) was given for pituitary desensitization from the mid-luteal phase in the previous cycle. Transvaginal ultrasound examination and serum estradiol measurement were then performed on Day 2–3 of the menstrual cycle. And urine-derived hMG (Lebaode, Lizhu, China) or recombinant FSH (Puregon, Organon, Dublin, Ireland or Gonal F, Merck Serono S.p.A, Modugno, Italy) was given at 150–225 IU per day based on the antral follicle count, age of women and previous ovarian response, according to the standard operation procedures of the center. Ovarian response was monitored by serial transvaginal scanning with or without hormonal monitoring. Further dosage adjustments were based on the ovarian response at the discretion of the clinicians in charge. For the antagonist protocol, patients were evaluated on Day 2–3 of the menstrual cycle and gonadotropins were administered afterwards. Antagonist 0.25 mg daily (Orgalutran, Organon, Dublin, Ireland) was given from the 6th day of ovarian stimulation until the day of ovulation trigger.

When three leading follicles reached ≥ 18 mm in diameter, hCG 10 000 IU (Lizhu, China) or Ovitrelle 250 µg (Merck Serono S.p.A., Modugno, Italy) was given to trigger final maturation of oocytes. Oocyte retrieval was performed around 36 h later.

### Fertilization and embryo evaluation and transfer

Semen samples were prepared by the swim-up procedure. About 2 h after oocyte retrieval, each oocyte was inseminated with approximately 20,000–30,000 motile spermatozoa. If the total number of motile sperm was < 10^5^ after washing or normal morphology was < 1%, intracytoplasmic sperm injection (ICSI) was performed. Oocytes were decoronated and checked for the presence of two pronuclei to confirm fertilization 24 h later. Embryos were graded on day 3 after retrieval as grade one to grade six according to the evenness of each blastomere and the percentage of fragmentation [11]. Embryos of 6–8 cells and of grade one or two were regarded as top-quality embryos. Some non-top-quality embryos were placed in extended culture until they reached the blastocyst stage.

A maximum of two embryos were transferred 3 days after the retrieval. Embryo transfer was performed by experienced clinicians. Excess good quality embryos were frozen for subsequent transfer.

### Randomization, intervention and blinding

On the day of embryo transfer, women were randomized into the atosiban (Tractocile, Ferring Pharmaceuticals, Kiel, Germany) or placebo groups in a 1 to 1 ratio according to a computer-generated randomization list. The number was placed in sealed envelopes, and opened by a nurse who was not involved in the study. Women in the atosiban group received intravenous administration of atosiban about 30 min before the transfer with a bolus dose of 6.75 mg over one minute (IRB of the hospital recommended to use smaller dose concerning the possible side effect of the atosiban). Those in the placebo group received only normal saline infusion for the same duration. In both the atosiban and placebo groups, women were medicated by syringes which looked identical and were prepared by a dedicated nurse in the center not involved in the study. Subjects, clinicians and laboratory staff were blinded to the group assignment. According to the standard operation procedures, all patients received oral and vaginal progesterone as the luteal phase support for 2 weeks. The codes for the treatment groups were revealed to the investigators only after the whole study and statistical analysis was completed.

### Pregnancy outcomes measures

The primary outcome measure was the live birth rate and the secondary outcome measures include positive pregnancy test, clinical pregnancy, ongoing pregnancy, miscarriage, multiple pregnancy and ectopic pregnancy rates. A baby born alive after 20 weeks gestation was classified as a live birth. Clinical pregnancy was defined as the presence of at least one gestational sac on ultrasound at 6 weeks. Ongoing pregnancy was defined as the presence of at least one fetus with heart pulsation on ultrasound beyond 8 weeks. Miscarriage rate was defined as the number of miscarriages before 20 weeks divided by the number of women with positive pregnancy test. Multiple pregnancy was defined as a pregnancy with more than one gestational sac detected on ultrasound at 6 weeks. Implantation rate was calculated as the number of gestational sacs seen on scanning divided by the number of embryos replaced. All pregnant women were followed up for the pregnancy outcome after delivery or miscarriage.

### Sample size calculation

The average live birth rate in the women with RIF of our center in 2016 was 20% per transfer. Assuming 20% increase in the clinical pregnancy rate to 40% after the use of atosiban, about 82 women in each arm were required at a power of 80% and a significance level of 5%. A total of 194 patients were recruited in this study to account for 15% drop-outs.

### Statistical analysis

Analysis was performed based on the intention-to-treat principle. Statistical comparisons were carried out using Mann–Whitney *U*-test, Chi-square test, Fisher’s exact test and Student *t*-test where appropriate with the Statistical Program for Social Sciences (SPSS, Version 20.0, Chicago, Illinois). Pearson correlation was used to analyze the association between serum estradiol (E_2_) level on the day of hCG with frequency of endometrial peristalsis on embryo transfer day. Logistic regression analysis was used to analyze factors predicting the live birth. A two-sided *P* < 0.05 was taken as statistically significant.

## Results

### Participant flow

**Fig. 1:**
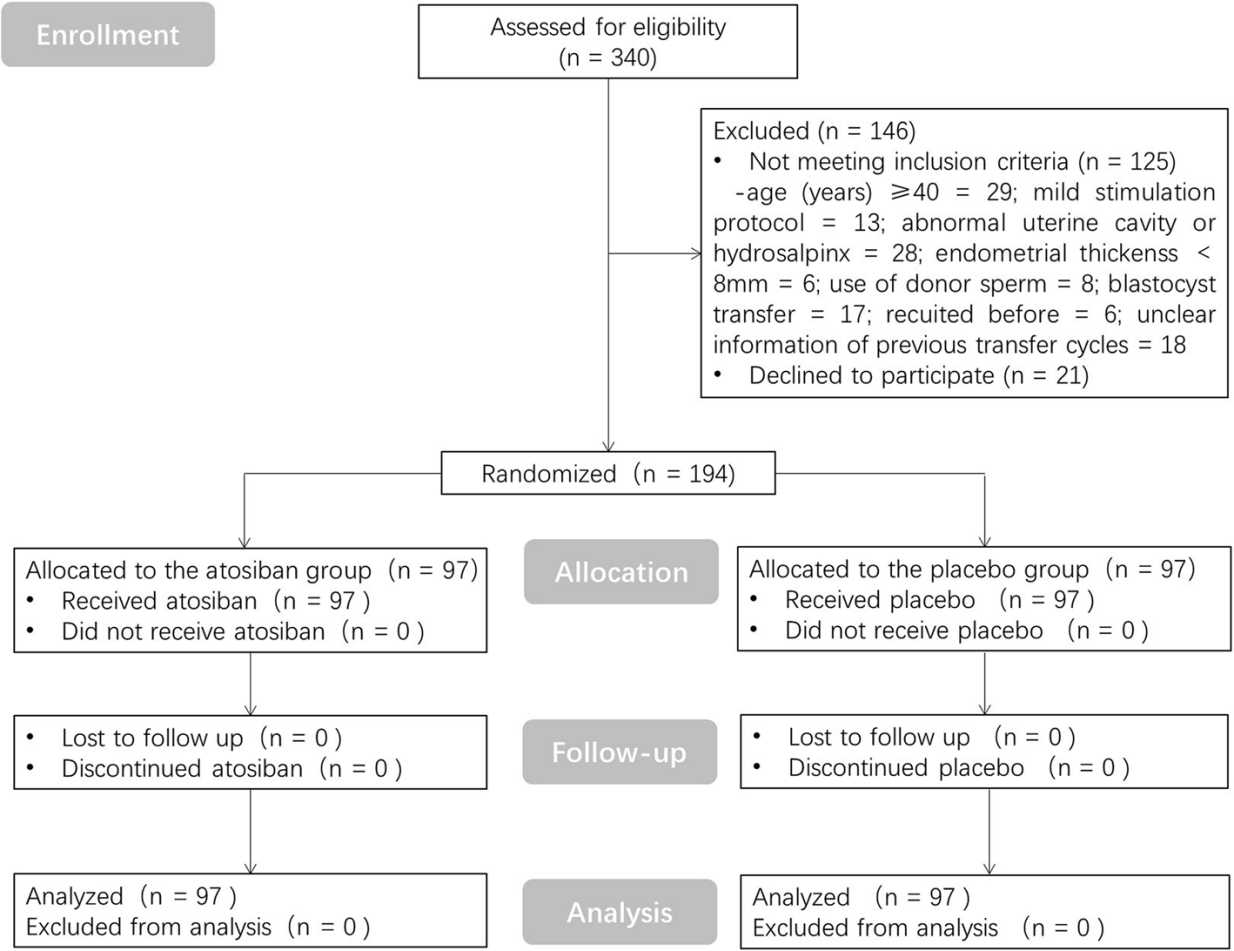
Flow chart of the randomized double blind study comparison of atosiban in patients with RIF

Between July 2017 and December 2019, 340 women were screened, 125 women did not meet the selection criteria and 21 women declined to participate (Fig. 1). Therefore, 194 women were finally recruited and 97 subjects were included in each group. None of them were lost to follow-up.

### Baseline and cycle characteristics

Baseline characteristics of the two groups are presented in Table 1. No significant differences were found with regard to age of women, BMI, antral follicle count, basal FSH, types and causes of infertility, the number of embryos previously transferred and number of previous failed embryo transfers.

**Table 1:** Demographic Comparison of atosiban group and placebo group

Characteristics	Atosiban (*n* = 97)	Placebo (*n* = 97)	*P*-value
Female age(years)	33(30–36)	33(30–36)	0.166
Body mass index (kg/m^2^)	22.0(19.9–24.6)	21.5(19.8–23.8)	0.205
Antral follicle count	12(9–19.5)	12(8–18)	0.796
FSH/HMG dosage (IU)	2025(1650–2700)	2025(1650–2512)	0.640
FSH/HMG duration (days)	10(8–11)	10(8–12)	0.863
Duration of infertility (years)	4(3–6)	4(3–6)	0.904
Types of infertility, n (%)			0.774
Primary infertility	51 (52.6)	49 (50.5)	
Secondary infertility	46 (47.4)	48 (59.5)	
Causes of infertility, n (%)			0.091
Tubal	49(50.5)	51 (52.6)	
Male factor	30 (30.9)	39 (40.2)	
Unexplained	5 (5.2)	1 (1)	
Mixed	13 (13.4)	6 (6.2)	
No. of embryos previously transferred	5(4–6)	4(4–6)	0.147
No. of previous failed embryo transfers	4(3–4)	3(3–4)	0.368
Stimulation protocol, n (%)			0.666
Long agonist	44(45.3)	47(48.4)	
Antagonist	53(54.6)	50(51.5)	
Insemination method, n (%)			0.774
IVF	45(46.4)	47(48.5)	
ICSI	52(53.6)	50(51.5)	
Estradiol on hCG day (pg/ml)	1898(1450–2552)	2025(1354–2658)	0.766
No. of oocytes obtained	8(6–11)	8(5–12)	0.803
No. of oocytes fertilized	5(3–8)	6(3–8)	0.524
No. of transferrable embryos	3(2–4)	3(2–4)	0.560
Total number of top-quality embryos	2(0–3)	2(1–3)	0.081
Endometrial thickness (mm)	11(9.6–12)	11(9–12)	0.762
No. of embryos transferred, n (%)			1.000
One	11 (11.3)	11 (11.3)	
Two	86 (88.7)	86 (88.7)	
Frequency of endometrial peristalsis (waves/min)	2(1.8–2.3)	2(1.5–2.3)	0.527

Note: Data are median (25^th^ and 75^th^ percentile) or number (percentage). Mann–Whitney *U*-test for continuous variables and chi-square test for categorical variables. There were no significant differences between groups

Stimulation protocol, insemination method, total FSH/HMG dosage /duration, serum E_2_ level on the day of hCG, endometrial thickness and the frequency of endometrial peristalsis on embryo transfer day were also comparable between two groups. The number of oocytes obtained, fertilized and number of top quality/transferable embryos and number of embryos transferred also showed no significant differences (Table 1).

### Pregnancy outcomes

There was no significant difference in the live birth rate between the atosiban and placebo groups (42.3% *vs* 35.1%, *P* = 0.302, RR = 1.206 (0.844–1.723)) (Table 2). No significant differences were found between the atosiban and two groups in the positive pregnancy test, clinical pregnancy, ongoing pregnancy, miscarriage, multiple pregnancy, ectopic pregnancy and implantation rates (Table 2).

**Table 2:** Comparison of pregnancy outcomes

	**Atosiban group**	**Placebo group**	***P*-value**	**Relative risk (95% CI)**
Positive pregnancy test rate	52.6% (51/97)	44.3% (43/97)	0.250	1.186(0.885–1.589)
Clinical pregnancy rate	50.5% (49/97)	42.3% (41/97)	0.249	1.195(0.881–1.621)
Ongoing pregnancy rate	42.3% (41/97)	35.1% (34/97)	0.302	1.206(0.844–1.723)
Live birth rate	42.3% (41/97)	35.1% (34/97)	0.302	1.206(0.844–1.723)
Miscarriage rate	15.7% (8/51)	16.3% (7/43)	0.938	0.964(0.380–2.441)
Multiple pregnancy rate	21.6% (11/51)	18.6% (8/43)	0.721	1.159(0.513–2.620)
Implantation rate	34.4% (63/183)	29.0% (53/183)	0.261	1.189(0.878–1.608)
Ectopic pregnancy rate	2% (1/51)	2.3% (1/43)	1	0.834(0.054–13.083)
Congenital abnormality rate	0	0		

Note: Data are percentage (number of positive finding/total number in the group). Chi-square test or Fisher’s exact test were used if appropriate. There were no significant differences between groups

### Subgroup analyses and logistic regression

**Fig. 2:**
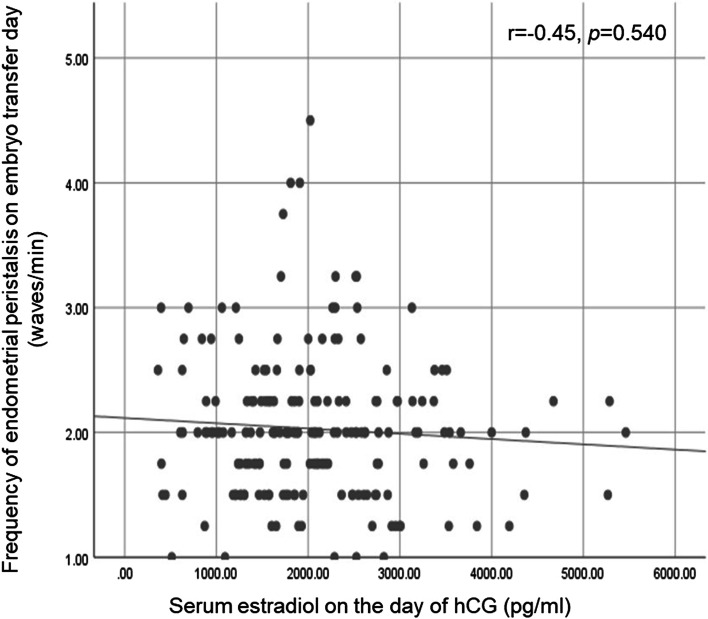
Correlation between the endometrial peristalsis on embryo transfer day and estradiol level on hCG day

Subgroup analysis was performed by stratifying women into the number of embryos previously transferred, number of previous failed embryo transfers, frequency of endometrial peristalsis on embryo transfer day (< 3 waves/min and ≥ 3 waves/min), and serum E_2_ level on the day of hCG above/below the median level (1906 pg/ml). The live birth rates in these subgroup analyses were also comparable between the atosiban and placebo groups (Table 3). There was no correlation between the serum E_2_ level on the day of hCG and the frequency of endometrial peristalsis on embryo transfer day (r = -0.45, *p* = 0.540) (Fig. 2).

**Table 3:** Subgroup analysis of the effect of atosiban on live birth rate

	**Atosiban**	**Placebo**	***P*-value**
No. of embryos previously transferred			
4	43.8% (21/48)	35.2% (19/54)	0.377
5	53.3% (8/15)	35.0% (7/20)	0.278
6	50.0% (8/16)	33.3% (4/12)	0.378
≥ 7	22.2% (4/18)	36.4% (4/11)	0.408
No. of previous failed embryo transfers			
3	44.4% (20/45)	37.3% (19/51)	0.474
4	51.5% (17/33)	33.3% (10/30)	0.145
5	30.8% (4/13)	41.7% (5/12)	0.571
≥ 6	0 (0/6)	0 (0/4)	
Frequency of endometrial peristalsis (waves/min)			
< 3	46.0% (40/87)	35.6% (32/90)	0.158
≥ 3	10.0% (1/10)	28.6% (2/7)	0.323
Estradiol on hCG day (pg/ml)			
< 1906	38.8% (19/49)	23.4% (11/47)	0.104
≥ 1906	45.8% (22/48)	46.0% (23/50)	0.987

Note: Data are percentage (number of live birth/total number in the group). Chi-square test or Fisher’s exact test were used if appropriate. There were no significant differences between groups

Binary logistic regression using the enter method was used to analyze the prediction for live birth in the fresh IVF cycle by the women’s age, duration and types of infertility, BMI, basal FSH, number of previous embryo transfers, stimulation protocol, insemination method, antral follicle count, FSH/HMG dosage and duration, estradiol level on hCG day, endometrial thickness and frequency of endometrial peristalsis on embryo transfer day, number of oocytes obtained, number of embryos replaced and atosiban/placebo. The frequency of endometrial peristalsis on embryo transfer day [Exp(B) 0.343, 95% CI 0.133–0.885; *P* = 0.027], total FSH/HMG dosage [Exp(B) 0.999, 95% CI 0.998–0.999; *P* = 0.002] and duration [Exp(B) 1.472, 95% CI 1.087–1.993; *P* = 0.012] were the significant factors which independently predicted the likelihood of a live birth, and use of atosiban was not associated with live birth following fresh embryo transfer in the women with RIF [Exp(B) 0.598, 95% CI 0.309–1.160; *P* = 0.128] (Table 4).

**Table 4:** Binary logistic regression analysis of factors predicting live birth

	***ꞵ***	**OR**	***P* value**	**95% CI for Exp(B)**	
				**Lower**	**Upper**
Female age	0.10	1.010	0.845	0.911	1.120
Duration of infertility	-0.059	0.943	0.421	0.817	1.088
Types of infertility	0.035	1.036	0.924	0.503	2.134
No. of previous embryo transfers	0.013	1.013	0.919	0.782	1.313
AFC	-0.019	0.981	0.510	0.928	1.038
Basal FSH	0.219	0.803	0.055	0.642	1.006
BMI	0.000	1.000	0.999	0.891	1.122
Stimulation protocol	0.513	1.701	0.223	0.723	4.001
Insemination method	0.755	2.128	0.089	0.892	5.074
Antral follicle count	-0.019	0.979	0.462	0.927	1.035
FSH/HMG dosage	-0.001	0.999	0.002	0.998	0.999
FSH/HMG duration	0.386	1.472	0.012	1.087	1.993
Use of atosiban	-0.593	0.533	0.088	0.280	1.092
No. of oocytes obtained	-0.044	0.996	0.938	0.897	1.106
No. of embryos replaced	0.407	1.503	0.562	0.379	5.961
Estradiol level on hCG day	0.000	1.000	0.401	0.999	1.000
Endometrial thickness	0.031	1.031	0.703	0.880	1.209
Frequency of endometrial peristalsis	-1.071	0.343	0.027	0.133	0.885

Note: All covariates in the table were adjusted in binary logistic regression model by enter method, adjusted odds ratios (ORs) and 95% CI were presented

## Discussion

To the best of our knowledge, this is the first randomized double-blind trial on the use of atosiban in RIF patients undergoing IVF. Our results did not show significant improvement in pregnancy outcomes, including the positive pregnancy test, implantation, clinical pregnancy, ongoing pregnancy and live birth rates in women receiving atosiban before the time of fresh embryo transfer.

Our results are in agreement with that of previous studies [10], which showed no benefit of IVF outcomes when using atosiban. A meta-analysis in 2016 revealed that the atosiban administration could only improve the embryo implantation rate but did not improve clinical pregnancy rate [12]. However some studies have reported beneficial effects of atosiban [6, 13, 14].The results of two meta-analysis in 2017 also showed that atosiban can significantly improve the implantation rate and clinical pregnancy rate of IVF, especially in RIF patients [15, 16]. However, only two studies [10, 13] included in these meta-analyses calculated the sample size before testing was performed and none of the studies has detected endometrial peristalsis during medication. Thus, poor methodological design of the included studies and inadequate analysis and sample size reduced the reliability of these findings.

Patients with RIF may have more uterine contractions [6, 7, 9]. Lan et al*.* [6] evaluated the frequency of uterine contractions by ultrasound in patients with RIF during the freeze–thaw embryo transfer cycle and found that the use of atosiban can reduce the frequency of endometrial peristaltic waves and obtain a better clinical pregnancy rate. Zhu et al. [17] reported the proportion of cycles with uterine contractions of > 3 waves/min was only 6.2% (18/292), but 65.0% (143/220) was found in the study of Fanchin et al*.* [4]. In the present study, the proportion of cycles with uterine contractions of ≥ 3 waves/min was 8.8% (17/194), which was in consistent with that in the study of Zhu et al. [17]. Our result suggested increased frequency of uterine contractions following ovarian stimulation were not often shown in patients with RIF and use of atosiban does not offer benefit if the proportion of women with uterine contractions ≥ 3/min constitutes a small proportion only. However, our subgroup analysis revealed no difference in the live birth rate between the two groups stratified by the frequency of endometrial peristalsis > 3 /min and ≤ 3/min. On the other hand, measuring uterine contractions was time consuming and had intra-variation between different observers, thus the accuracy of the measurement is questionable. We did not measure endometrial peristalsis after embryo transfer and compare uterine contractions before and after atosiban administration in the present study due to ethical reasons and the use of atosiban unlikely offers benefit if the effect of this drug on reduction uterine contractions is limited.

The cause of RIF can be attributed to the two main factors, namely the dysfunction of the embryo and the endometrium [18]. With the development of ART, it is less difficult to obtain high-quality embryos. Therefore, endometrial receptivity has become a key factor for the success of embryo transfer [19]. During the ovarian stimulation cycle, the super-physiological estradiol concentration could induce endometrial production of oxytocin, formation of oxytocin receptors and indirectly synthesis/release of prostaglandin (PG) F2a, and thus may affect the endometrial receptivity [20, 21]. However, our subgroup analysis indicated that serum estradiol level in stimulated IVF cycles was not correlated with frequency of endometrial peristalsis on embryo transfer day, which was consistent with previous study [4, 22]. In fact, the effect of high levels of estrogen on pregnancy outcome is still controversial. Some researchers considered that high serum E_2_ levels are not detrimental to IVF results [23, 24]. In present study, no significant correlation was found between pregnancy outcomes and serum estradiol level on the day of hCG. Although we found the frequency of endometrial peristalsis was negatively associated with live birth following fresh embryo transfer, the cause of RIF other than excessive uterine contractions in this group of patients still needs to be investigated. Aneuploidy leads to the majority of preclinical pregnancy losses and is therefore a likely cause of RIF in many cases, especially in women of advanced age [25]. However, we did not test embryos aneuploidy status by PGT-A. As the result, such RIF patients with possibly implantation of aneuploidy embryos were not excluded in this study and this might have impact on our results.

Another reason why we did not observe any benefit of atosiban may be related to the regimen of atosiban infusion used in the present study, which was based on the study of Chou et al. [14]. Atosiban is a very short acting drug and was administered 30 min before the transfer with a bolus dose of 6.75 mg over one minute intravenously. This dosage is different from that in some of the studies [10] which reported atosiban was given intravenously for 1 h around embryo transfer and continued infusion for another 2 h after embryo transfer with the total administered dose 37.5 mg. Although there is no consensus on the optimal dose and exposure time of atosiban to exert the maximal effect, the duration of medication was shortened and the reduction of the total dose in this study may attribute to no benefit effect of atosiban. Moreover, even if the atosiban really work on reducing frequency and amplitude of uterine contractions, it may not last long enough after stopping the atosiban infusion to produce appreciable effects on the outcome measures. Since implantation take place 3 days after cleavage embryos transfer, a prolonged atosiban infusion over 1–2 days or a maintenance therapy using oral non-steroidal anti-inflammatory therapy after the atosiban infusion may be associated with a sustained reduction in uterine contractions after embryo transfer, thereby may led to a higher live birth rate. Further suitable dose finding studies are warranted to answer these questions.

One limitation of this study was the relatively small sample size, which aimed to detect an increase of live birth rate from 20 to 40% after atosiban infusion. Although not reached statistic difference, our data showed around 7% (42.3% *vs* 35.1%) difference of live birth rate between the atosiban and placebo group. It might be a greater difference on the live birth rate with more patients included. Thus, a multi-center RCT with larger sample size would be needed in the future to verify our findings.

As for the safety of atosiban, Pierzynski et al. [26] showed that atosiban did not affect the survival of single-cell rabbit embryos or decrease the percentage of hatched rabbit blastocysts and had no adverse influence on human sperm motility. The common side effects of atosiban are digestive system symptom [27]. A meta-analysis also confirmed that atosiban resulted in fewer maternal side-effects than nifidipine, with no difference in pregnancy prolongation [28]. There is currently a lack of evidence that atosiban has embryotoxic effect. We did not find congenital abnormalities in the newborns in either group of current study.

## Conclusions

In conclusion, the use of atosiban given before fresh embryo transfer did not improve the live birth rate in RIF patients. Similar results were found stratified by the number of embryos previously transferred, number of previous failed embryo transfers, frequency of endometrial peristalsis on embryo transfer day (≥ 3 waves/min) or serum estradiol on the day of hCG above the median level. The clinical value of using atosiban need to be further studied.

## Data Availability

All data generated or analyzed in this study are included in this published article.

## Abbreviations

IVF-ET: In vitro fertilization and embryo transfer
RIF: Recurrent implantation failure
ART: Assisted reproductive technology
RCT: Randomized controlled trial
GnRH: Gonadotropin-releasing hormone
hCG: Human chorionic gonadotropin
BMI: Body mass index
FSH: Follicle stimulating hormone
E_2_: Estradiol
HMG: Human menopausal gonadotropin
PG: Prostaglandin
